# Potential neural substrates underlying circadian and olfactory disruptions in preclinical Alzheimer’s disease

**DOI:** 10.3389/fnins.2023.1295998

**Published:** 2023-11-29

**Authors:** Quiana L. Jeffs, Jonathan F. Prather, William D. Todd

**Affiliations:** Department of Zoology and Physiology, Program in Neuroscience, University of Wyoming, Laramie, WY, United States

**Keywords:** circadian dysfunction, olfactory dysfunction, Alzheimer’s disease, olfactory bulb, suprachiasmatic nucleus, biomarker, dopamine, vasoactive intestinal peptide

## Abstract

Alzheimer’s disease (AD) is the leading cause of dementia, with over 45 million patients worldwide, and poses significant economic and emotional burdens to both patients and caregivers, significantly raising the number of those affected. Unfortunately, much of the existing research on the disease only addresses a small subset of associated symptomologies and pathologies. In this review, we propose to target the earliest stages of the disease, when symptomology first arises. In these stages, before the onset of hallmark symptoms of AD such as cognitive impairments and memory loss, circadian and olfactory disruptions arise and are detectable. Functional similarities between circadian and olfactory systems provide a basis upon which to seek out common mechanisms in AD which may target them early on in the disease. Existing studies of interactions between these systems, while intriguing, leave open the question of the neural substrates underlying them. Potential substrates for such interactions are proposed in this review, such as indirect projections that may functionally connect the two systems and dopaminergic signaling. These substrates may have significant implications for mechanisms underlying disruptions to circadian and olfactory function in early stages of AD. In this review, we propose early detection of AD using a combination of circadian and olfactory deficits and subsequent early treatment of these deficits may provide profound benefits to both patients and caregivers. Additionally, we suggest that targeting research toward the intersection of these two systems in AD could uncover mechanisms underlying the broader set of symptoms and pathologies that currently elude researchers.

## Introduction

Alzheimer’s disease (AD) is the leading cause of dementia, with over 45 million patients worldwide, constituting between 10 and 30% of individuals above the age of 65 (Masters et al., 2015; Prince et al., 2015). As of 2021, AD was the seventh leading cause of death in the US overall, and the fifth leading cause in individuals over the age of 65 (Alzheimer’s Association, 2022). Many therapies available for patients merely mediate symptoms and do not slow the progression of the disease, more often than not leading to institutionalization – which places significant financial and emotional burden on both patients and caregivers (Masters et al., 2015; Alzheimer’s Association, 2022). Patients over the age of 65 make up over 95% of the patient population, although patients with an early-onset subtype of AD may begin to experience symptoms as early as the age of 35 (van der Flier et al., 2011; Prince et al., 2015). Late-onset AD thus accounts for the vast majority of cases, typically emerging around the ages of 65 or above.

This discrepancy in patients experiencing early- and late-onset AD, among other factors, presents significant hurdles to understanding the underlying mechanisms of this disease. The vast majority of identified genetic markers for the disease are associated with early-onset AD, while most cases of late-onset AD are sporadic in origin (van der Flier et al., 2011). It is not clear why AD-related molecular pathologies spread in the stereotypical spatiotemporal pattern that they follow, nor is it clear which, if any, of those pathologies are related to factors governing the onset of AD symptoms (Benilova et al., 2012; Sengoku, 2020). Further, it is an open question whether these identified pathologies even cause the symptoms and eventual neurodegeneration observed in AD or are instead coincidental with other hallmarks of the disease and aging in general such as neuroinflammation and oxidative stress (Benilova et al., 2012; Calsolaro and Edison, 2016; Ionescu-Tucker and Cotman, 2021; Bai et al., 2022). These wide-ranging barriers to fully characterizing AD highlight the importance of determining where to begin in the search for causal factors underlying it.

One reasonable approach for investigating causal factors is to begin with a focus on the earliest disturbances associated with AD. Disruptions to olfactory and circadian function occur in the preclinical stages of AD, before the onset of hallmark symptoms such as cognitive impairment and memory loss (Bacon et al., 1998; Wilson et al., 2009; Musiek et al., 2018). Olfactory and circadian dysfunction alike are highly prevalent among AD patients, making their examination relevant to the broader patient population (Attems et al., 2005; Leng et al., 2019). Olfactory disruption in aged individuals is strongly predictive of the onset of AD, sometimes preceding the clinical presentation of the disease by at least a year (Wilson et al., 2009; Devanand et al., 2015). It is possible that the mechanisms underlying such olfactory dysfunction may be causative for the further progression of AD. Even if this is not the case, the early appearance of such disruptions supports their use as a predictive clinical biomarker and warrants an investigation into their potentially shared mechanisms with AD onset and progression. Similarly, circadian disruption in aged individuals is strongly predictive of the onset of AD, sometimes also preceding the clinical presentation of the disease by a substantial amount of time (Musiek et al., 2018). These disruptions in themselves have been proposed as a causative mechanism in the onset of AD and the buildup of the molecular pathologies associated with the disease (Roh et al., 2014; Kress et al., 2018). This possibility and the early presentation of circadian disruption additionally warrant its use as a predictive clinical biomarker and further investigation of its role in mechanisms of AD onset and progression. Early detection of AD through the use of these combinatorial biomarkers is of vital importance for potentially slowing the onset of the disease, and giving patients and caregivers the opportunity to plan for the future while patients remain lucid (Rasmussen and Langerman, 2019).

In this review we argue for the use of olfactory and circadian disruptions as predictive clinical biomarkers for the onset of AD, and we propose that these systems should be investigated in tandem regarding the mechanisms underlying AD onset and progression. We also discuss the early onset of AD-related olfactory and circadian disturbances, and the functional similarities between these two systems. We consider the well-documented interactions between the olfactory and circadian systems which have yet to be thoroughly characterized mechanistically. Finally, we explore potential mechanisms which may underlie these interactions, highlighting those which seem most plausible and may have most relevance to early stages of AD.

## Alzheimer’s disease

AD is characterized by the buildup of two misfolded proteins: amyloid-beta (Aβ), which accumulates extracellularly as plaques, and hyperphosphorylated tau (pTau), which accumulates intracellularly as neurofibrillary tangles. The temporospatial spread of these pathologies follows a fairly stereotypical pattern, beginning in the entorhinal cortex and subcortical areas and then spreading throughout the rest of the brain (Braak and Braak, 1991; Schonheit et al., 2004). Of particular note, olfactory areas, including the entorhinal cortex, and brainstem structures that project to the circadian system, such as the lateral parabrachial nucleus, consistently develop AD pathology earlier than other brain regions (Rub et al., 2001; Braak et al., 2006; Warfield et al., 2023), which may underlie the early appearance of dysfunction of these processes. Researchers debate the contributions of plaques and tangles to the symptoms of AD, although many studies agree that the load of Aβ and pTau correlates with the severity of symptoms (Nelson et al., 2012; Attems et al., 2014). As the disease becomes more severe, plaques and tangles continue to spread throughout the brain, and eventually lead to neurodegeneration (i.e., massive cell death).

While AD was first described over one hundred years ago, it remains an extremely elusive disease, with no clear cause for its onset, and disagreement regarding the effects of its associated molecular pathologies (Hippius and Neundorfer, 2003). It has been argued that the presence of plaques and tangles may be purely correlative with the symptoms of the disease rather than causative, as other pathological hallmarks present, namely neuroinflammation and oxidative stress, have been identified as key contributors to symptoms of AD (Kinney et al., 2018; Long and Holtzman, 2019; Bai et al., 2022). In both laboratory and clinical studies, drugs which clear Aβ have unfortunately not been found to stop disease progression or ameliorate symptoms, lending credence to the idea that this pathological marker may be coincidental (Small and Duff, 2008; Panza et al., 2019). Another barrier to understanding this disease is the animal models that are used in basic research settings. Late-onset AD comprises over 95% of the total patient population and is overwhelmingly sporadic in origin (Bettens et al., 2010). While risk factors such as sex, diabetes, and heart disease have been identified, there are no clear causes for sporadic AD (Scheyer et al., 2018; Silva et al., 2019). Early-onset AD, in contrast, is tied more clearly to genetic predisposition; these genetic mutations are the basis upon which animal models, most often transgenic mice, are created (Yokoyama et al., 2022). Although the genetic basis for these animal models may differ from the cause of disease onset for a large majority of the AD patient population, many AD animal models mirror the anatomical spread of pathology and the temporal progression of many behavioral and physiological symptoms observed in AD patients. This parallel between patterns of progression in animal models and humans makes AD animal models a valuable tool in basic research for investigating the disease (Yokoyama et al., 2022). Importantly for this review, several of these animal models have been found to specifically exhibit olfactory and circadian symptoms similar to those observed in AD patients (Sheehan and Musiek, 2020; Zhang et al., 2022).

### Preclinical olfactory and circadian symptoms

Symptomatically, olfactory and circadian dysfunction are often the earliest detectable signs of AD. These deficits appear well before the onset of AD’s hallmark symptoms of cognitive impairment and memory loss and may in fact contribute to disease onset (Bacon et al., 1998; Devanand et al., 2008; Doty, 2009; Wilson et al., 2009; Musiek et al., 2018). Olfactory and circadian processes become weaker with normal aging (Doty and Kamath, 2014; Hood and Amir, 2017), but this dysfunction is significantly more pronounced in age-matched AD patients (Mesholam et al., 1998; Masurkar and Devanand, 2014; Wang et al., 2015; Musiek et al., 2018).

Early olfactory deficits associated with AD are typically confined to those that rely on the perception of an odor’s identity rather than odor detection. In longitudinal studies linking olfactory dysfunction with the onset of AD, odor identification and discrimination (the ability to detect differences in odors) are more impaired than sensitivity (Rahayel et al., 2012; Jung et al., 2019). The nature of these deficits may elucidate the mechanisms underlying them, such as a differential impact on downstream olfactory structures at this stage of this disease, causing perceptual deficits in identifying and differentiating odors while leaving the detection of odors intact. It is difficult to determine the proportions of preclinical AD patients with each of these forms of disruption as the majority of validated clinical assessments assess only one facet of olfactory function.

Early circadian deficits associated with AD are typically confined to shifts and fragmentation of rhythms, suggesting an issue with entrainment to the daily light cycle. These present as phase delays, defined as rhythms shifted to be later in the day, and sleep fragmentation, in which patients sleep for small bouts rather than during one consolidated period (Tranah et al., 2011; Musiek et al., 2018). Circadian disruptions have been found in basic research settings to have a bidirectional relationship with AD severity, in that pathologies associated with AD lead to circadian disruptions (Sterniczuk et al., 2010; Duncan et al., 2012; Warfield et al., 2023), while circadian disruptions lead to increases in pathological load (Roh et al., 2014; Kress et al., 2018). One potential mechanism for the increase in pathological load as a result of circadian disruption may be related to the glymphatic system, which has been found to aid in the clearance of Aβ during sleep, particularly during long, consolidated bouts of sleep (Kang et al., 2009; Xie et al., 2013; Hablitz et al., 2020).

The early emergence of olfactory and circadian disruptions in AD and the high proportion of patients who experience them strongly suggest that it would be clinically useful to assess the function of these systems in tandem as a predictive clinical biomarker. There are a small number of validated tests that are utilized for the purpose of assessing olfactory function as a biomarker for AD (Doty et al., 1984, 1996; Hummel et al., 1997; Jackman and Doty, 2005). Although circadian function has been strongly suggested for use as a biomarker, there does not yet exist a validated test of circadian function for this purpose (Musiek et al., 2015; Hoyt and Obrietan, 2022). Such a test could be as simple as providing an actigraphy watch that records periods of rest and activity to at-risk individuals in order to assess times and length of rest.

## Functional similarities of circadian and olfactory systems

The olfactory and circadian centers of the brain exhibit strong functional similarities involving the generation of intrinsic genetic rhythmicity and the synchronization of such rhythmicity in downstream structures. Cells throughout the brain and body express oscillator genes that include Clock, Bmal1, Per, and Cry. In short, Clock and Bmal1 induce the expression of Per and Cry genes, which then are translated into proteins that subsequently feedback and inhibit their own expression by Clock and Bmal1. This negative feedback loop takes about 24 h to complete, providing the foundation for circadian rhythmicity (Reppert and Weaver, 2002). On a cellular scale, these rhythms impact the relative levels of metabolic function across the day (Huang et al., 2011). On an organismal scale, these rhythms modulate physiological processes ranging from sleep to regulation of body temperature to the ability to detect a sensory stimulus (Saper, 2013). The vast majority of cells in the body require an input to synchronize these rhythms with those of other cells, and historically this synchronizing input was thought to come solely from the suprachiasmatic nucleus (SCN) of the hypothalamus, the “master pacemaker” and hub of the circadian system (Reppert and Weaver, 2002). More recently, evidence has emerged that a subset of neural olfactory tissues does not require the SCN as a synchronizing input (Granados-Fuentes et al., 2004a, 2006). Instead, those olfactory sites receive their synchronizing input from the olfactory bulb (OB), the hub of the olfactory system (Granados-Fuentes et al., 2006).

Within the SCN, rhythmic expression of oscillator genes is entrained, or synchronized by, light-related activity transmitted from the retina to the SCN via a direct axonal projection (Reppert and Weaver, 2002). This entrainment to light allows diurnal organisms to align the onset of activity each day to the light cycle, and nocturnal organisms to align the onset of activity to the dark cycle. Pacemaker neurons within the SCN receive this entraining input and use the timing of light activity to rhythmically express the neuropeptide vasoactive intestinal peptide (VIP). Expression of VIP is necessary for maintaining synchrony of the expression of oscillator genes among SCN cells as well as in cells peripheral to the SCN (Aton et al., 2005; Todd et al., 2020). The SCN also receives input from brain regions other than the retina which send signals that can also act as entraining cues, and these timing-related cues are known as zeitgebers (Hastings et al., 1998; Saper, 2013). These zeitgebers drive rhythmic activity in the SCN, and that activity synchronizes the activity in downstream pathways and peripheral tissues, including the olfactory system.

Within the OB, cells are synchronously rhythmic in their expression of oscillatory genes (Granados-Fuentes et al., 2004b). Cells in the OB appear to receive input from the SCN as OB rhythms are entrained by SCN activity, although the exact pathway for this is unknown (Granados-Fuentes et al., 2004a). Importantly, however, this input is not necessary to maintain synchronous rhythmic gene expression in OB neurons (Granados-Fuentes et al., 2004a). Cells elsewhere in the body are also affected by the synchronizing signal of the SCN, but those cells do not maintain synchronized rhythmicity without that influence from the SCN. Thus, the OB is intrinsically rhythmic in its gene expression, and the purpose of the input from the SCN is to preserve synchrony between activity of the OB and that of cells elsewhere throughout the body. As in the case of the SCN, rhythmic VIP expression within the OB is necessary for maintaining synchrony of the expression of oscillator genes among OB cells (Miller et al., 2014). Also similar to the SCN, the OB appears to receive entraining signals that can act as zeitgebers from structures aside from the SCN (Nolasco et al., 2012). It is unclear how the OB sends synchronizing signals to structures peripheral to it, but it has been shown that it communicates such signals to downstream olfactory structures, namely the piriform cortex (Granados-Fuentes et al., 2006; Takeuchi et al., 2023). Studies of mice have demonstrated that these rhythms are not merely incidental and have significant behavioral implications for olfactory sensitivity and discrimination, as measured by neural activity and behavior, as well as more complex behavioral tasks requiring olfaction (Nordin et al., 2003; Granados-Fuentes et al., 2011; Pantazopoulos et al., 2011; Takeuchi et al., 2023).

These functional similarities between the circadian and olfactory systems provide a basis upon which to seek out common mechanisms in AD which may target them during the early stages of the disease. Based on these similarities, it is possible that such mechanisms may target pathways connecting the two systems, the function of the neuropeptide VIP, the expression of oscillatory genes, or regions which project to both the SCN and the OB. More work is needed to examine how AD pathology impacts the function of such olfactory and circadian processes and leads to symptomology, and whether targeting shared pathways can ameliorate such comorbid disruptions.

## Interactions between circadian and olfactory systems

The interactions between circadian and olfactory systems are bidirectional and wide-ranging, with direct consequences for symptomology present in AD. As discussed in the previous section, the circadian system exerts its influence on the olfactory system by synchronizing rhythms of the OB to those of the rest of the body. Importantly, the SCN is not necessary to synchronize these rhythms within the OB or its immediate downstream structures, as demonstrated by research in rodents (Granados-Fuentes et al., 2011). Much of the literature regarding interactions between these systems suggests that the olfactory system exerts a robust influence on the circadian system by providing a timing cue complementary to light input. Relevant to AD, when olfactory input is removed, circadian deficits similar to those present in early stages of AD occur as detailed below.

Much work suggests that olfactory stimulation has a profound effect on circadian timing, which has strong implications for associations between olfactory and circadian deficits present in preclinical AD. One study performed in the nocturnal primate *Microcebus murinus* found that olfactory bulbectomy led to delays in re-entrainment to altered light cycles as well as changes to rhythms of body temperature and locomotor activity (Perret et al., 2003). This experiment is particularly interesting as it represents the only one of its kind to examine the effect of a gross olfactory perturbation on circadian function in a primate. Additionally, such circadian disruption that results from the removal of olfactory input indicates problems with entrainment similar to those seen in early AD patients. Translational support for this result is present in several other studies in rodents that led to similar observations of disruptions in several circadian rhythms after partial or full lesions to the OB, including stress responses, sleep, hormone release, and immune responses (Marcilhac et al., 1997; Saulea et al., 1998; Vinkers et al., 2009; Yuan et al., 2020). Timed presentation of odors, conversely, has an opposite effect of enhancing entrainment of circadian rhythms to light. Presentation at regular times of both social and non-social odorants in rodents has been observed to not only increase rates of re-entrainment to altered light cycles, but also to enhance activation of the immediate early gene cFos in the SCN in response to light pulses (Amir et al., 1999; Governale and Lee, 2001; Jechura et al., 2006). Amir and colleagues demonstrated enhanced cFos activation within the SCN in response to olfactory stimulation, which is compelling evidence for a potential pathway that carries olfactory information to the circadian system, with an intriguing caveat. In this study, rats were either exposed to only olfactory stimulation, a light pulse simultaneously with olfactory stimulation, or a light pulse only, during the late rest period or early active period. Rats exposed to stimuli during the late rest period did not experience any significant phase shifts, and rats exposed to stimuli during the early active period only displayed phase shifts modulated by olfactory stimulation when combined with a light pulse. These phase shifts were enhanced as compared to light pulses alone. Similarly, cFos activation in the SCN was absent in the case of olfactory stimulation alone, but cFos activation was enhanced when olfactory stimulation was combined with a light pulse as compared to light pulses alone (Amir et al., 1999). These results suggest that properly timed olfactory stimuli can accelerate reentrainment. Although such experiments have yet to be recapitulated in humans, there is good evidence for a role of repeatedly providing stimulation by the same set of odors in olfactory rehabilitation in individuals with olfactory loss (Hummel et al., 2009; Pieniak et al., 2022). Of consequence to AD patients, this may support that bright light therapy combined with strong olfactory stimulation in the early morning could provide the dual benefit of rescuing light entrainment deficits and olfactory deficits.

These studies, while intriguing, leave open the question of neural substrates that underlie these interactions. In the next section of this review, we discuss potential substrates for these interactions, although in all cases these substrates require further study to determine their validity.

## Potential mechanisms underlying interactions between systems

**Figure 1:**
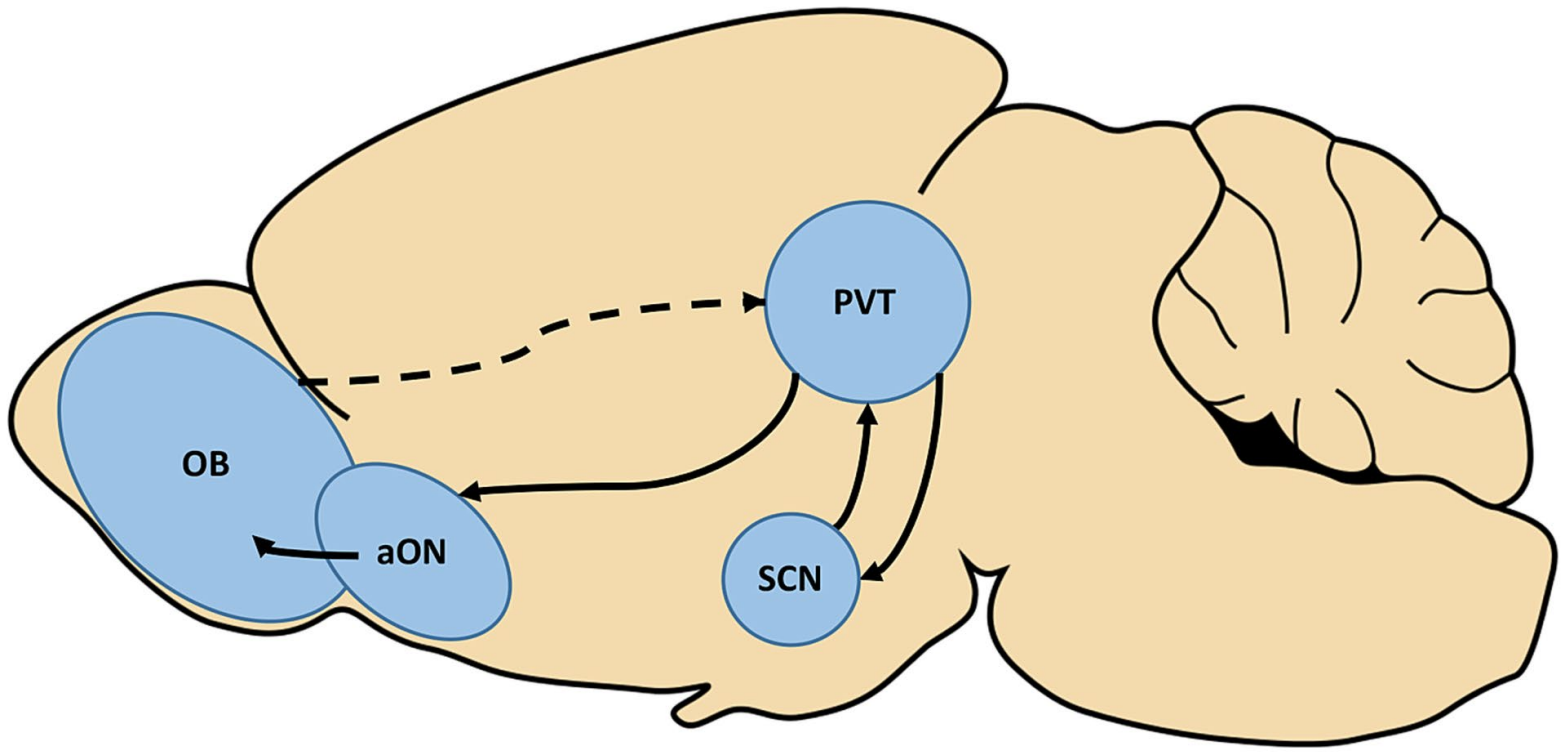
The paraventricular thalamus (PVT) represents a potential, bidirectional relay between the olfactory and circadian systems. Olfactory stimulation has been found to induce activation of the PVT, although the pathway which carries this information is not yet known (dotted line). The PVT is also a major source of input to the suprachiasmatic nucleus (SCN) of the hypothalamus, the hub of the circadian system. Conversely, the SCN is known to project to the PVT, which has been shown to further send direct projections to the anterior olfactory nucleus (aON). Completing this potential circuit, the aON is a source of input to the olfactory bulb (OB).

The observed interactions between circadian and olfactory systems strongly suggest the presence of an indirect pathway conferring information between them. While some early work utilizing nonspecific tracing methods suggested there may be direct projections between these systems, more recent studies utilizing cell-type specific retrograde tracing have not recapitulated this (Krout et al., 2002; Todd et al., 2020). However, many inputs to the SCN follow indirect pathways so this may be the case here as well. As depicted in Figure 1, one possible pathway between the SCN to olfactory structures relies on a relay, the paraventricular thalamus (PVT). Odor presentations have been shown to induce cFos expression in the PVT, which is a major source of input to the SCN (Moga and Moore, 1997; Amir et al., 1999). However, the pathway which would bring odor information to this area is unclear. Intriguingly, the SCN also sends projections to the PVT which then projects directly to several olfactory regions including the anterior olfactory nucleus (Moga et al., 1995). This pathway with the PVT as a relay may represent a single, bidirectional route through which circadian and olfactory information is sent between the systems.

In addition to this proposed pathway, there exist several confirmed pathways linking the olfactory system and hypothalamus specifically for the purpose of sharing information about environmental odors and reproduction (Gascuel et al., 2012). In particular, the nervus terminalis (also known as the overlooked cranial nerve “0” which is present medial to the olfactory nerve) is of interest. The nervus terminalis, described in humans as well as other mammalian literature, projects to both olfactory and hypothalamic structures, namely the preoptic area and infundibulum, and may play a bidirectional, modulatory role between these areas (Vilensky, 2014). It is worth noting that existing research has heavily implicated the nervus terminalis in reproductive development, particularly in regard to the hypothalamic–pituitary-gonad axis (Vilensky, 2014). However, given that a comprehensive description of the functions of this oft-overlooked cranial nerve is still an active area of research, further studies will be needed to determine its significance in regard to the circadian system.

These proposed pathways represent potential therapeutic targets by which to ameliorate symptoms and pathology of these two systems targeted in early AD. Changes to cell signaling or cell counts of these regions have garnered little attention in AD research, so further studies are needed to determine what form these therapies would take. Neuropeptides shared between circadian and olfactory systems may also underlie their interactions, or may represent a shared substrate that is similarly targeted in early stages of AD. VIP is expressed in regions associated with these systems and is crucial for the proper functioning of each system (Miller et al., 2014; Todd et al., 2020). Additionally, dopamine is released in response to olfactory stimulation while the circadian system expresses dopamine receptors, which may underlie entrainment to reward (Grippo et al., 2017; Gillman et al., 2019; Midroit et al., 2021).

VIP is a prime candidate as a shared molecular target of circadian and olfactory systems in AD as its rhythmic expression in these areas is necessary for synchronizing the oscillatory gene activity of cells within them (Aton et al., 2005; Miller et al., 2014). Of note, inactivation of VIP cells in the OB has been found in mice to impair odor detection and discrimination and may also impair outbound signaling from the OB, meaning that global impairment of VIP function would strongly impact both normal circadian and olfactory function (Wang et al., 2022). Accordingly, a study utilizing a mouse model in which VIP-expressing cells were targeted for deletion of the protein kinase mTOR found that both circadian and olfactory functioning was significantly disrupted (Liu et al., 2018). This should be of particular interest to AD researchers as mTOR signaling has also been implicated in learning and memory, and, more specifically, appears to have a bidirectional upregulatory relationship with Aβ, leading to an increase in hyperphosphorylation of Tau (Hoeffer et al., 2008; Oddo, 2012; Huang et al., 2013). Additionally, mTOR signaling has been found to contribute to inflammation and oxidative stress which have been postulated to be the source of symptoms associated with AD (Rapaka et al., 2022). Rapamycin, a drug which inhibits mTOR signaling, has been found to significantly reduce the load of Aβ and slow or halt the progression of symptoms in an AD mouse model and has been suggested as a clinical treatment for AD patients (Spilman et al., 2010; Kaeberlein and Galvan, 2019). Further research is needed to determine if such downregulation by rapamycin specifically rescues AD-related circadian and olfactory deficits, and how early in the course of AD mTOR is dysregulated. If it is dysregulated early, mTOR dysfunction may underlie a wide host of pathological disturbances present in AD.

**Figure 2:**
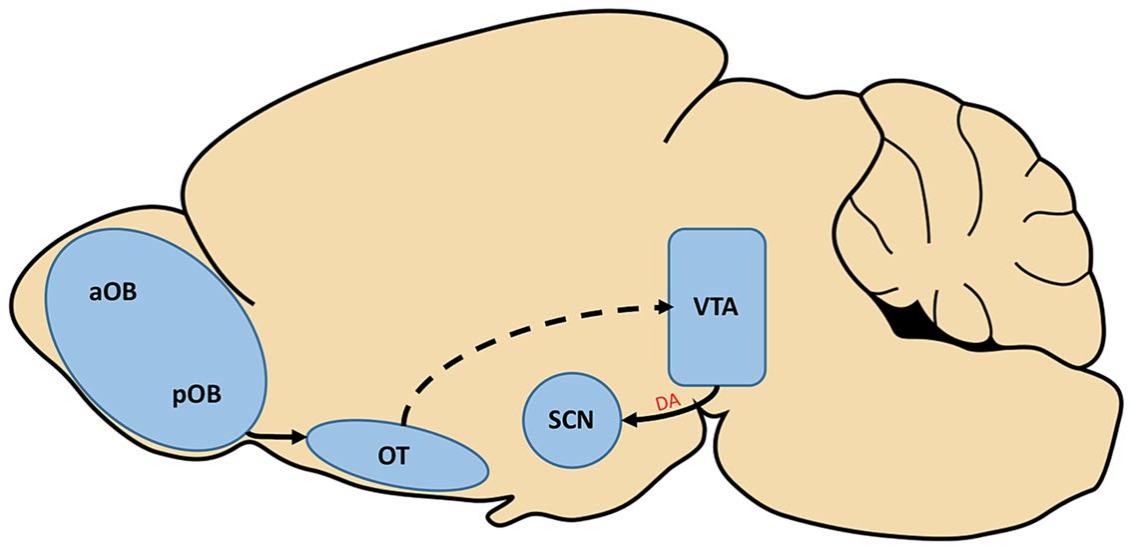
The posterior olfactory bulb (pOB) is known to selectively send information regarding pleasant odorants through projections to the olfactory tubercle (OT). The activation of the olfactory system by pleasant odorants has also been found to activate cells of the ventral tegmental area (VTA), although the pathway which carries this information is not yet confirmed (dotted line). In a separate study, the VTA was found to send direct dopaminergic input (A) to the suprachiasmatic nucleus (SCN) of the hypothalamus, the hub of the circadian system. aOB, anterior olfactory bulb.

An especially intriguing idea is that dopamine may represent both a shared substrate for symptomology of AD and a neuropeptide that mediates interactions between the circadian and olfactory systems. The hedonic nature of olfaction suggests a potential causal link between the release of dopamine in response to pleasant odors and entrainment of the SCN. Thus, subsequent removal of this dopamine cue would then lead to disruptions in entrainment of circadian rhythms. Reward in itself can act as a zeitgeber, providing important complementary timing information to the SCN in conjunction with light (Gillman et al., 2019). A study performed in rodents and partially validated in humans suggests that attractive odors can serve as a reward, activating dopaminergic neurons in the ventral tegmental area (VTA) (Midroit et al., 2021). This finding suggests that in day-to-day activities, timed presentation of pleasant odors such as the aroma of brewing coffee may cause the timed release of dopamine which could act as a zeitgeber. As depicted in Figure 2, there is also a circuit basis for this interaction. Midroit and colleagues demonstrated that attractive odors are processed selectively in the posterior OB, and these signals are then sent directly to the olfactory tubercle, which then presumably projects to the VTA based on its activation by attractive odors. Intriguingly, the VTA sends direct dopaminergic input to the SCN that is received by the D1 receptor subtype on SCN neurons (Grippo et al., 2017). Further validating this as a potential functional circuit between the olfactory and circadian systems, the effects of directly stimulating dopamine signaling from the VTA to the SCN are strikingly similar to those observed in studies of timed administration of odorants in animal studies. Both timed direct stimulation of dopaminergic VTA cells and timed administration of odorants have been found to increase the rate of re-entrainment to shifts in light cycles (Amir et al., 1999; Governale and Lee, 2001; Jechura et al., 2006; Grippo et al., 2017). Together, these findings provide both functional and anatomical evidence in support for a circuit from olfactory to circadian centers with the dopaminergic VTA as a relay. Relevant to AD, some recent studies have found a reduction of dopaminergic activity within the VTA in early stages of AD in both mouse models and humans that may underlie deficits in reward processing and memory (Nobili et al., 2017; De Marco and Venneri, 2018). It is conceivable that, in addition to underlying these deficits, this loss may also contribute to entrainment deficits of the SCN as a result of the removal or weakening of reward signals such as odors.

## Discussion

AD represents an excessively complex and multi-faceted problem to tackle, and understanding the mechanisms of its onset is a logical place to start. To this end, shedding light on the processes underlying interactions between the systems affected earliest in the disease may make this solution even more attainable. Evidence presented in this review demonstrates that the circadian and olfactory systems are an excellent place to start in this endeavor. Circadian and olfactory deficits are often the earliest detectable signs of AD, which alone is sufficient for their use as predictive clinical biomarkers for AD. Studies clearly demonstrate that these systems share significant functional similarities and interact bidirectionally. Further, the disruption of such interactions leads to circadian deficits similar to those present in early AD. Given this evidence, it follows that studying AD in the context of both the circadian and olfactory systems may yield insights of consequence to the broader symptomology of the disease.

Molecular pathologies associated with AD consistently develop in higher olfactory regions, such as the entorhinal cortex, and regions that project to the circadian system, such as the lateral parabrachial nucleus of the brainstem earlier than other brain regions (Rub et al., 2001; Braak et al., 2006; Warfield et al., 2023). These pathologies may or may not directly contribute to symptomology associated with early deficits of these systems, but nonetheless are strong evidence for their early targeting by mechanisms of AD. Deficits in the function of these systems arise significantly earlier than the ability to clinically diagnose the disease, lending benefit to their use as predictive biomarkers for disease onset (Bacon et al., 1998; Devanand et al., 2008; Doty, 2009; Wilson et al., 2009; Musiek et al., 2018). Particularly in the case of circadian deficits, this dysfunction may, in fact, contribute to the onset of AD (Roh et al., 2014; Kress et al., 2018), emphasizing the importance of closely observing individuals with circadian deficits who are at risk of developing AD.

The SCN and OB, hubs of the circadian and olfactory systems, respectively, both display the unique feature of intrinsically-generated rhythmicity of clock genes (Reppert and Weaver, 2002; Granados-Fuentes et al., 2004b). In each case, this rhythmicity is entrained by external cues, and, in turn, the rhythmic signals produced in each area project to downstream regions to provide an entraining signal (Hastings et al., 1998; Granados-Fuentes et al., 2004a, 2006; Nolasco et al., 2012; Saper, 2013). The entraining signals provided by each of these systems have significant implications for behavior, and the rhythms that they provide have been found to be negatively impacted in AD, particularly in the case of the circadian system (Granados-Fuentes et al., 2011; Saper, 2013; Musiek et al., 2018; Takeuchi et al., 2023). Synchrony of rhythmicity within the cells of the SCN and OB is dependent on the neuropeptide VIP, which is a potential shared target by mechanisms of AD (Aton et al., 2005; Oddo, 2012; Miller et al., 2014; Liu et al., 2018; Todd et al., 2020).

Strengthening the case for a connection between the circadian and olfactory systems, the two systems are well-established to interact with one another. While not solely necessary for its entrainment, the SCN does provide an entraining signal to the OB (Granados-Fuentes et al., 2011). The olfactory system similarly exerts a significant influence on the circadian system that allows for odor cues to provide a complementary entraining signal to light cues (Amir et al., 1999; Governale and Lee, 2001; Perret et al., 2003; Jechura et al., 2006). Olfactory deficits in preclinical AD may thus underlie or contribute to problems with entrainment of the circadian system including phase delays and sleep fragmentation. In turn, rescuing early olfactory deficits through therapies such as odor training, i.e., repeatedly providing stimulation by the same set of odors, may help to mitigate early circadian deficits (Hummel et al., 2009; Pieniak et al., 2022).

Two distinct indirect pathways that may underlie these interactions are proposed in this review, one relying on the PVT as a relay and the other on the nervus terminalis as a relay. As depicted in Figure 1, in the case of the PVT this pathway may be bidirectional, with odor information activating the PVT which then directly projects to the SCN (Moga and Moore, 1997; Amir et al., 1999). The SCN also sends projections to the PVT which in turn projects directly to several olfactory regions (Moga et al., 1995). In the case of the nervus terminalis, this pathway may also be bidirectional, as the nervus terminalis projects to both olfactory and hypothalamic structures (Vilensky, 2014). Unfortunately, due to the lack of research regarding this oft-overlooked cranial nerve, it is not yet known whether there exist direct projections carrying olfactory or circadian information to the nervus terminalis.

Most relevant to AD, two molecular mechanisms that may underlie interactions between the circadian and olfactory systems are additionally proposed in this review, one being the shared expression of VIP in the SCN and OB and the other being dopaminergic signaling as a result of olfactory stimulation within the SCN. VIP is necessary for proper functioning of circadian and olfactory processes (Aton et al., 2005; Miller et al., 2014; Wang et al., 2022), and accordingly, at least one study involving the deletion of the protein kinase mTOR in VIP led to both circadian and olfactory deficits (Liu et al., 2018). mTOR has additionally been implicated in several other processes that are impaired in AD, meaning that mTOR dysregulation may represent a mechanism of AD which targets a broad range of systems disrupted in the disease (Hoeffer et al., 2008; Oddo, 2012; Huang et al., 2013).

Dopaminergic cells of the VTA are activated in response to odor stimulation, and the VTA is separately known to send direct dopaminergic input to the SCN (Grippo et al., 2017; Midroit et al., 2021). Strong evidence for this as a functional, dopaminergic pathway is the fact that the entrainment effects of odor stimulation on the SCN are strikingly similar to those of dopaminergic release from VTA cells on the SCN (Amir et al., 1999; Governale and Lee, 2001; Jechura et al., 2006; Grippo et al., 2017), as depicted in Figure 2. Implicating this pathway as a target of AD, dopaminergic signaling has previously been found to be reduced in the VTA in early stages of the disease (Nobili et al., 2017; De Marco and Venneri, 2018). This reduced signaling may underlie deficits to circadian entrainment in this stage of AD as it would reduce odor stimulation to the SCN which has been shown to produce entrainment deficits in rodent and primate studies (Marcilhac et al., 1997; Perret et al., 2003; Vinkers et al., 2009).

The combined use of circadian and olfactory deficits as a predictive biomarker for the onset of AD represents a robust, practical, and economically feasible method to screen for individuals at risk for developing the disease. Circadian deficits can be examined in patients through the use of an actigraphy watch, which measures times of rest and activity (Wang et al., 2015), and these are widely used, and relatively inexpensive and accessible. Similarly, several tests already exist for the examination of olfactory deficits that do not require extensive training to be administered correctly, and can be accessed relatively easily by clinicians (Doty et al., 1984, 1996; Hummel et al., 1997; Jackman and Doty, 2005). Incorporating actigraphy and olfactory testing into longitudinal studies using analyses that detect preclinical AD, such as cerebrospinal fluid biomarkers or amyloid beta and tau positron emission tomography imaging (Musiek et al., 2018), could reveal shared neural mechanisms that lead to better understanding of disease progression.

In our discussion of research findings presented throughout this review, it is important to note that many come from rodent studies which utilize mouse models that express genetic mutations derived from individuals with early-onset AD (Yokoyama et al., 2022). Early-onset AD patients constitute less than 5 % of the whole AD patient population, so it is conceivable that these models do not represent the properties of the disease shared by the vast majority of patients (Bettens et al., 2010). However, properties such as the spatiotemporal spread of molecular pathologies throughout the brain and the progression of symptomology are recapitulated in many AD mouse models, lending credence to their use in preclinical work on AD (Yokoyama et al., 2022). Such models thus represent an opportunity to identify and manipulate the specific neural circuits that develop AD-related dysfunction associated with this pathology and its temporal relationship with the emergence of olfactory and circadian dysfunction, which is much less feasible in preclinical AD patients.

We have presented evidence demonstrating the early onset of circadian and olfactory deficits in relation to the onset of AD, which in conjunction with the similarities and interactions of these two systems, may represent shared processes that are among the earliest of targets of the disease. This points to a need in the field of preclinical AD research for researchers with multidisciplinary training who adopt a holistic approach of examining deficits in each of these systems in tandem, rather than researchers would focus only on one or the other. More specifically, this necessitates the establishment and use of AD mouse models that exhibit both circadian and olfactory deficits similar those seen in AD patients. As it stands, this field of research includes many studies that are limited in scope and yield similarly myopic results, providing little insight into overall disease mechanisms that target multiple systems. However, we argue that taking an integrative and multidisciplinary approach to preclinical AD research that incorporates a focus on the earliest comorbid disruptions, such as that seen in circadian and olfactory processing, has the potential to lead to broader insights into the disease as a whole.

## Conclusion

Currently, several mechanisms for the disruption of circadian and olfactory deficits in AD have been proposed (Cedernaes et al., 2017; Misiak et al., 2017; Lachen-Montes et al., 2019; Liu et al., 2023). Unfortunately, many of these mechanisms fail to explain or address the deficits in each of these systems that are comorbid at the clinical onset of the disease. Further, many of these mechanisms similarly fail to address the vast pathology and symptomology of the disease. At present, many of the studies in AD research address its mechanistic underpinnings from a single-faceted approach, such as determining molecular changes to a particular system or neural structure in AD that may lead to its associated deficits. Instead, we have argued here that an integrative approach to AD research that incorporates both circadian and olfactory function may uncover broader mechanisms that not only affect these systems, but that globally contribute to the onset and progression of the disease.

## Author contributions

QJ: Writing – original draft, Writing – review & editing. JP: Writing – original draft, Writing – review & editing. WT: Writing – original draft, Writing – review & editing.

## References

[ref2] Amir S. Cain S. Sullivan J. Robinson B. Stewart J. (1999). Olfactory stimulation enhances light-induced phase shifts in free-running activity rhythms and Fos expression in the suprachiasmatic nucleus. Neuroscience 92, 1165–1170. doi: 10.1016/s0306-4522(99)00222-5, PMID: 10426475

[ref3] Alzheimer’s Association (2022). 2022 Alzheimer's disease facts and figures. Alzheimers Dement. 18, 700–789. doi: 10.1002/alz.12638, PMID: 35289055

[ref4] Aton S. J. Colwell C. S. Harmar A. J. Waschek J. Herzog E. D. (2005). Vasoactive intestinal polypeptide mediates circadian rhythmicity and synchrony in mammalian clock neurons. Nat. Neurosci. 8, 476–483. doi: 10.1038/nn1419, PMID: 15750589 PMC1628303

[ref5] Attems J. Lintner F. Jellinger K. A. (2005). Olfactory involvement in aging and Alzheimer's disease: an autopsy study. J. Alzheimers Dis. 7, 149–157. doi: 10.3233/jad-2005-7208 15851853

[ref6] Attems J. Walker L. Jellinger K. A. (2014). Olfactory bulb involvement in neurodegenerative diseases. Acta Neuropathol. 127, 459–475. doi: 10.1007/s00401-014-1261-7 24554308

[ref7] Bacon A. W. Bondi M. W. Salmon D. P. Murphy C. (1998). Very early changes in olfactory functioning due to Alzheimer's disease and the role of apolipoprotein E in olfaction. Ann. N. Y. Acad. Sci. 855, 723–731. doi: 10.1111/j.1749-6632.1998.tb10651.x 9929677

[ref8] Bai R. Guo J. Ye X. Y. Xie Y. Xie T. (2022). Oxidative stress: the core pathogenesis and mechanism of Alzheimer's disease. Ageing Res. Rev. 77:101619. doi: 10.1016/j.arr.2022.101619, PMID: 35395415

[ref9] Benilova I. Karran E. De Strooper B. (2012). The toxic Abeta oligomer and Alzheimer's disease: an emperor in need of clothes. Nat. Neurosci. 15, 349–357. doi: 10.1038/nn.3028, PMID: 22286176

[ref10] Bettens K. Sleegers K. Van Broeckhoven C. (2010). Current status on Alzheimer disease molecular genetics: from past, to present, to future. Hum. Mol. Genet. 19, R4–R11. doi: 10.1093/hmg/ddq142, PMID: 20388643 PMC2875058

[ref11] Braak H. Alafuzoff I. Arzberger T. Kretzschmar H. Del Tredici K. (2006). Staging of Alzheimer disease-associated neurofibrillary pathology using paraffin sections and immunocytochemistry. Acta Neuropathol. 112, 389–404. doi: 10.1007/s00401-006-0127-z, PMID: 16906426 PMC3906709

[ref12] Braak H. Braak E. (1991). Neuropathological stageing of Alzheimer-related changes. Acta Neuropathol. 82, 239–259. doi: 10.1007/BF00308809, PMID: 1759558

[ref13] Calsolaro V. Edison P. (2016). Neuroinflammation in Alzheimer's disease: current evidence and future directions. Alzheimers Dement. 12, 719–732. doi: 10.1016/j.jalz.2016.02.010, PMID: 27179961

[ref14] Cedernaes J. Osorio R. S. Varga A. W. Kam K. Schioth H. B. Benedict C. (2017). Candidate mechanisms underlying the association between sleep-wake disruptions and Alzheimer's disease. Sleep Med. Rev. 31, 102–111. doi: 10.1016/j.smrv.2016.02.002, PMID: 26996255 PMC4981560

[ref15] De Marco M. Venneri A. (2018). Volume and connectivity of the ventral tegmental area are linked to neurocognitive signatures of Alzheimer's disease in humans. J. Alzheimers Dis. 63, 167–180. doi: 10.3233/JAD-171018, PMID: 29578486

[ref16] Devanand D. P. Lee S. Manly J. Andrews H. Schupf N. Doty R. L.. (2015). Olfactory deficits predict cognitive decline and Alzheimer dementia in an urban community. Neurology 84, 182–189. doi: 10.1212/WNL.0000000000001132, PMID: 25471394 PMC4336090

[ref17] Devanand D. P. Liu X. Tabert M. H. Pradhaban G. Cuasay K. Bell K.. (2008). Combining early markers strongly predicts conversion from mild cognitive impairment to Alzheimer's disease. Biol. Psychiatry 64, 871–879. doi: 10.1016/j.biopsych.2008.06.020, PMID: 18723162 PMC2613777

[ref18] Doty R. L. (2009). The olfactory system and its disorders. Semin. Neurol. 29, 074–081. doi: 10.1055/s-0028-1124025 19214935

[ref19] Doty R. L. Kamath V. (2014). The influences of age on olfaction: a review. Front. Psychol. 5:20. doi: 10.3389/fpsyg.2014.00020, PMID: 24570664 PMC3916729

[ref20] Doty R. L. Marcus A. Lee W. W. (1996). Development of the 12-item cross-cultural smell identification test (CC-SIT). Laryngoscope 106, 353–356. doi: 10.1097/00005537-199603000-00021 8614203

[ref21] Doty R. L. Shaman P. Dann M. (1984). Development of the University of Pennsylvania Smell Identification Test: a standardized microencapsulated test of olfactory function. Physiol. Behav. 32, 489–502. doi: 10.1016/0031-9384(84)90269-5, PMID: 6463130

[ref22] Duncan M. J. Smith J. T. Franklin K. M. Beckett T. L. Murphy M. P. St Clair D. K.. (2012). Effects of aging and genotype on circadian rhythms, sleep, and clock gene expression in APPxPS1 knock-in mice, a model for Alzheimer's disease. Exp. Neurol. 236, 249–258. doi: 10.1016/j.expneurol.2012.05.011 22634208

[ref23] Gascuel J. Lemoine A. Rigault C. Datiche F. Benani A. Penicaud L.. (2012). Hypothalamus-olfactory system crosstalk: orexin a immunostaining in mice. Front. Neuroanat. 6:44. doi: 10.3389/fnana.2012.00044, PMID: 23162437 PMC3492705

[ref24] Gillman A. G. Rebec G. V. Pecoraro N. C. Kosobud A. E. K. (2019). Circadian entrainment by food and drugs of abuse. Behav. Process. 165, 23–28. doi: 10.1016/j.beproc.2019.05.017, PMID: 31132444 PMC6703158

[ref25] Governale M. M. Lee T. M. (2001). Olfactory cues accelerate reentrainment following phase shifts and entrain free-running rhythms in female *Octodon degus* (Rodentia). J. Biol. Rhythm. 16, 489–501. doi: 10.1177/074873001129002169, PMID: 11669422

[ref26] Granados-Fuentes D. Ben-Josef G. Perry G. Wilson D. A. Sullivan-Wilson A. Herzog E. D. (2011). Daily rhythms in olfactory discrimination depend on clock genes but not the suprachiasmatic nucleus. J. Biol. Rhythm. 26, 552–560. doi: 10.1177/0748730411420247, PMID: 22215613 PMC3658462

[ref27] Granados-Fuentes D. Prolo L. M. Abraham U. Herzog E. D. (2004a). The suprachiasmatic nucleus entrains, but does not sustain, circadian rhythmicity in the olfactory bulb. J. Neurosci. 24, 615–619. doi: 10.1523/JNEUROSCI.4002-03.2004, PMID: 14736846 PMC6729269

[ref28] Granados-Fuentes D. Saxena M. T. Prolo L. M. Aton S. J. Herzog E. D. (2004b). Olfactory bulb neurons express functional, entrainable circadian rhythms. Eur. J. Neurosci. 19, 898–906. doi: 10.1111/j.0953-816x.2004.03117.x, PMID: 15009137 PMC3474850

[ref29] Granados-Fuentes D. Tseng A. Herzog E. D. (2006). A circadian clock in the olfactory bulb controls olfactory responsivity. J. Neurosci. 26, 12219–12225. doi: 10.1523/JNEUROSCI.3445-06.2006, PMID: 17122046 PMC6675419

[ref30] Grippo R. M. Purohit A. M. Zhang Q. Zweifel L. S. Guler A. D. (2017). Direct midbrain dopamine input to the suprachiasmatic nucleus accelerates circadian entrainment. Curr. Biol. 27, 2465–2475.e3. doi: 10.1016/j.cub.2017.06.084, PMID: 28781050 PMC5568465

[ref31] Hablitz L. M. Pla V. Giannetto M. Vinitsky H. S. Staeger F. F. Metcalfe T.. (2020). Circadian control of brain glymphatic and lymphatic fluid flow. Nat. Commun. 11:4411. doi: 10.1038/s41467-020-18115-2, PMID: 32879313 PMC7468152

[ref32] Hastings M. H. Duffield G. E. Smith E. J. Maywood E. S. Ebling F. J. (1998). Entrainment of the circadian system of mammals by nonphotic cues. Chronobiol. Int. 15, 425–445. doi: 10.3109/07420529808998700 9787934

[ref33] Hippius H. Neundorfer G. (2003). The discovery of Alzheimer's disease. Dialogues Clin. Neurosci. 5, 101–108. doi: 10.31887/DCNS.2003.5.1/hhippius, PMID: 22034141 PMC3181715

[ref34] Hoeffer C. A. Tang W. Wong H. Santillan A. Patterson R. J. Martinez L. A.. (2008). Removal of FKBP12 enhances mTOR-raptor interactions, LTP, memory, and perseverative/repetitive behavior. Neuron 60, 832–845. doi: 10.1016/j.neuron.2008.09.037, PMID: 19081378 PMC2630531

[ref35] Hood S. Amir S. (2017). The aging clock: circadian rhythms and later life. J. Clin. Invest. 127, 437–446. doi: 10.1172/JCI90328, PMID: 28145903 PMC5272178

[ref36] Hoyt K. R. Obrietan K. (2022). Circadian clocks, cognition, and Alzheimer's disease: synaptic mechanisms, signaling effectors, and chronotherapeutics. Mol. Neurodegener. 17:35. doi: 10.1186/s13024-022-00537-9, PMID: 35525980 PMC9078023

[ref37] Huang W. Ramsey K. M. Marcheva B. Bass J. (2011). Circadian rhythms, sleep, and metabolism. J. Clin. Invest. 121, 2133–2141. doi: 10.1172/JCI46043, PMID: 21633182 PMC3104765

[ref38] Huang W. Zhu P. J. Zhang S. Zhou H. Stoica L. Galiano M.. (2013). mTORC2 controls actin polymerization required for consolidation of long-term memory. Nat. Neurosci. 16, 441–448. doi: 10.1038/nn.3351, PMID: 23455608 PMC3615448

[ref39] Hummel T. Rissom K. Reden J. Hahner A. Weidenbecher M. Huttenbrink K. B. (2009). Effects of olfactory training in patients with olfactory loss. Laryngoscope 119, 496–499. doi: 10.1002/lary.20101 19235739

[ref40] Hummel T. Sekinger B. Wolf S. R. Pauli E. Kobal G. (1997). Sniffin' sticks': olfactory performance assessed by the combined testing of odor identification, odor discrimination and olfactory threshold. Chem. Senses 22, 39–52. doi: 10.1093/chemse/22.1.39, PMID: 9056084

[ref41] Ionescu-Tucker A. Cotman C. W. (2021). Emerging roles of oxidative stress in brain aging and Alzheimer's disease. Neurobiol. Aging 107, 86–95. doi: 10.1016/j.neurobiolaging.2021.07.014, PMID: 34416493

[ref42] Jackman A. H. Doty R. L. (2005). Utility of a three-item smell identification test in detecting olfactory dysfunction. Laryngoscope 115, 2209–2212. doi: 10.1097/01.mlg.0000183194.17484.bb, PMID: 16369168

[ref43] Jechura T. J. Mahoney M. M. Stimpson C. D. Lee T. M. (2006). Odor-specific effects on reentrainment following phase advances in the diurnal rodent, *Octodon degus*. Am. J. Physiol. Regul. Integr. Comp. Physiol. 291, R1808–R1816. doi: 10.1152/ajpregu.00005.2006, PMID: 16840658

[ref44] Jung H. J. Shin I. S. Lee J. E. (2019). Olfactory function in mild cognitive impairment and Alzheimer's disease: a meta-analysis. Laryngoscope 129, 362–369. doi: 10.1002/lary.27399, PMID: 30565695

[ref45] Kaeberlein M. Galvan V. (2019). Rapamycin and Alzheimer's disease: time for a clinical trial? Sci. Transl. Med. 11:eaar4289. doi: 10.1126/scitranslmed.aar4289, PMID: 30674654 PMC6762017

[ref46] Kang J. E. Lim M. M. Bateman R. J. Lee J. J. Smyth L. P. Cirrito J. R.. (2009). Amyloid-beta dynamics are regulated by orexin and the sleep-wake cycle. Science 326, 1005–1007. doi: 10.1126/science.1180962, PMID: 19779148 PMC2789838

[ref47] Kinney J. W. Bemiller S. M. Murtishaw A. S. Leisgang A. M. Salazar A. M. Lamb B. T. (2018). Inflammation as a central mechanism in Alzheimer's disease. Alzheimers Dement (N Y) 4, 575–590. doi: 10.1016/j.trci.2018.06.014, PMID: 30406177 PMC6214864

[ref48] Kress G. J. Liao F. Dimitry J. Cedeno M. R. FitzGerald G. A. Holtzman D. M.. (2018). Regulation of amyloid-beta dynamics and pathology by the circadian clock. J. Exp. Med. 215, 1059–1068. doi: 10.1084/jem.20172347, PMID: 29382695 PMC5881473

[ref49] Krout K. E. Kawano J. Mettenleiter T. C. Loewy A. D. (2002). CNS inputs to the suprachiasmatic nucleus of the rat. Neuroscience 110, 73–92. doi: 10.1016/s0306-4522(01)00551-6, PMID: 11882374

[ref50] Lachen-Montes M. Gonzalez-Morales A. Palomino M. Ausin K. Gomez-Ochoa M. Zelaya M. V.. (2019). Early-onset molecular derangements in the olfactory bulb of Tg2576 mice: novel insights into the stress-responsive olfactory kinase dynamics in Alzheimer's disease. Front. Aging Neurosci. 11:141. doi: 10.3389/fnagi.2019.00141, PMID: 31244650 PMC6579864

[ref51] Leng Y. Musiek E. S. Hu K. Cappuccio F. P. Yaffe K. (2019). Association between circadian rhythms and neurodegenerative diseases. Lancet Neurol. 18, 307–318. doi: 10.1016/S1474-4422(18)30461-7, PMID: 30784558 PMC6426656

[ref52] Liu W. Ma R. Sun C. Xu Y. Liu Y. Hu J.. (2023). Implications from proteomic studies investigating circadian rhythm disorder-regulated neurodegenerative disease pathology. Sleep Med. Rev. 70:101789. doi: 10.1016/j.smrv.2023.101789, PMID: 37253318

[ref53] Liu D. Stowie A. de Zavalia N. Leise T. Pathak S. S. Drewes L. R.. (2018). mTOR signaling in VIP neurons regulates circadian clock synchrony and olfaction. Proc. Natl. Acad. Sci. U. S. A. 115, E3296–E3304. doi: 10.1073/pnas.1721578115, PMID: 29555746 PMC5889665

[ref54] Long J. M. Holtzman D. M. (2019). Alzheimer disease: an update on pathobiology and treatment strategies. Cells 179, 312–339. doi: 10.1016/j.cell.2019.09.001, PMID: 31564456 PMC6778042

[ref55] Marcilhac A. Maurel D. Anglade G. Ixart G. Mekaouche M. Hery F.. (1997). Effects of bilateral olfactory bulbectomy on circadian rhythms of ACTH, corticosterone, motor activity and body temperature in male rats. Arch. Physiol. Biochem. 105, 552–559. doi: 10.1076/apab.105.6.552.3273 9587645

[ref56] Masters C. L. Bateman R. Blennow K. Rowe C. C. Sperling R. A. Cummings J. L. (2015). Alzheimer's disease. Nat. Rev. Dis. Primers. 1:15056. doi: 10.1038/nrdp.2015.56 27188934

[ref57] Masurkar A. V. Devanand D. P. (2014). Olfactory dysfunction in the elderly: basic circuitry and alterations with Normal aging and Alzheimer's disease. Curr Geriatr Rep 3, 91–100. doi: 10.1007/s13670-014-0080-y, PMID: 25045620 PMC4097327

[ref58] Mesholam R. I. Moberg P. J. Mahr R. N. Doty R. L. (1998). Olfaction in neurodegenerative disease: a meta-analysis of olfactory functioning in Alzheimer's and Parkinson's diseases. Arch. Neurol. 55, 84–90. doi: 10.1001/archneur.55.1.84 9443714

[ref59] Midroit M. Chalencon L. Renier N. Milton A. Thevenet M. Sacquet J.. (2021). Neural processing of the reward value of pleasant odorants. Curr. Biol. 31, 1592–1605.e9. doi: 10.1016/j.cub.2021.01.066, PMID: 33607032 PMC9291255

[ref60] Miller J. E. Granados-Fuentes D. Wang T. Marpegan L. Holy T. E. Herzog E. D. (2014). Vasoactive intestinal polypeptide mediates circadian rhythms in mammalian olfactory bulb and olfaction. J. Neurosci. 34, 6040–6046. doi: 10.1523/JNEUROSCI.4713-13.2014, PMID: 24760863 PMC3996221

[ref61] Misiak M. Vergara Greeno R. Baptiste B. A. Sykora P. Liu D. Cordonnier S.. (2017). DNA polymerase beta decrement triggers death of olfactory bulb cells and impairs olfaction in a mouse model of Alzheimer's disease. Aging Cell 16, 162–172. doi: 10.1111/acel.12541, PMID: 27686631 PMC5242308

[ref62] Moga M. M. Moore R. Y. (1997). Organization of neural inputs to the suprachiasmatic nucleus in the rat. J. Comp. Neurol. 389, 508–534. doi: 10.1002/(sici)1096-9861(19971222)389:3<508::aid-cne11>3.0.co;2-h, PMID: 9414010

[ref63] Moga M. M. Weis R. P. Moore R. Y. (1995). Efferent projections of the paraventricular thalamic nucleus in the rat. J. Comp. Neurol. 359, 221–238. doi: 10.1002/cne.903590204 7499526

[ref64] Musiek E. S. Bhimasani M. Zangrilli M. A. Morris J. C. Holtzman D. M. Ju Y. S. (2018). Circadian rest-activity pattern changes in aging and preclinical Alzheimer disease. JAMA Neurol. 75, 582–590. doi: 10.1001/jamaneurol.2017.4719, PMID: 29379963 PMC5885197

[ref65] Musiek E. S. Xiong D. D. Holtzman D. M. (2015). Sleep, circadian rhythms, and the pathogenesis of Alzheimer disease. Exp. Mol. Med. 47:e148. doi: 10.1038/emm.2014.121, PMID: 25766617 PMC4351409

[ref66] Nelson P. T. Alafuzoff I. Bigio E. H. Bouras C. Braak H. Cairns N. J.. (2012). Correlation of Alzheimer disease neuropathologic changes with cognitive status: a review of the literature. J. Neuropathol. Exp. Neurol. 71, 362–381. doi: 10.1097/NEN.0b013e31825018f7 22487856 PMC3560290

[ref67] Nobili A. Latagliata E. C. Viscomi M. T. Cavallucci V. Cutuli D. Giacovazzo G.. (2017). Dopamine neuronal loss contributes to memory and reward dysfunction in a model of Alzheimer's disease. Nat. Commun. 8:14727. doi: 10.1038/ncomms14727 28367951 PMC5382255

[ref68] Nolasco N. Juarez C. Morgado E. Meza E. Caba M. (2012). A circadian clock in the olfactory bulb anticipates feeding during food anticipatory activity. PLoS One 7:e47779. doi: 10.1371/journal.pone.0047779, PMID: 23094084 PMC3477144

[ref69] Nordin S. Lotsch J. Murphy C. Hummel T. Kobal G. (2003). Circadian rhythm and desensitization in chemosensory event-related potentials in response to odorous and painful stimuli. Psychophysiology 40, 612–619. doi: 10.1111/1469-8986.00062 14570168

[ref70] Oddo S. (2012). The role of mTOR signaling in Alzheimer disease. Front. Biosci. (Schol. Ed.) S4, 941–952. doi: 10.2741/s310, PMID: 22202101 PMC4111148

[ref71] Pantazopoulos H. Dolatshad H. Davis F. C. (2011). A fear-inducing odor alters PER2 and c-Fos expression in brain regions involved in fear memory. PLoS One 6:e20658. doi: 10.1371/journal.pone.0020658, PMID: 21655193 PMC3105109

[ref72] Panza F. Lozupone M. Logroscino G. Imbimbo B. P. (2019). A critical appraisal of amyloid-beta-targeting therapies for Alzheimer disease. Nat. Rev. Neurol. 15, 73–88. doi: 10.1038/s41582-018-0116-6 30610216

[ref73] Perret M. Aujard F. Seguy M. Schilling A. (2003). Olfactory bulbectomy modifies photic entrainment and circadian rhythms of body temperature and locomotor activity in a nocturnal primate. J. Biol. Rhythm. 18, 392–401. doi: 10.1177/0748730403254248, PMID: 14582855

[ref74] Pieniak M. Oleszkiewicz A. Avaro V. Calegari F. Hummel T. (2022). Olfactory training - thirteen years of research reviewed. Neurosci. Biobehav. Rev. 141:104853. doi: 10.1016/j.neubiorev.2022.104853, PMID: 36064146

[ref9001] Prince M. Wimo A. Guerchet M. Ali G. C. Wu Y. T. Prina M. (2015). World Alzheimer Report 2015. The Global Impact of Dementia: An Analysis of Prevalence, Incidence, Cost and Trends. London, UK: Alzheimer\u0027s Disease International (ADI).

[ref75] Rahayel S. Frasnelli J. Joubert S. (2012). The effect of Alzheimer's disease and Parkinson's disease on olfaction: a meta-analysis. Behav. Brain Res. 231, 60–74. doi: 10.1016/j.bbr.2012.02.047, PMID: 22414849

[ref76] Rapaka D. Bitra V. R. Challa S. R. Adiukwu P. C. (2022). mTOR signaling as a molecular target for the alleviation of Alzheimer's disease pathogenesis. Neurochem. Int. 155:105311. doi: 10.1016/j.neuint.2022.105311, PMID: 35218870

[ref77] Rasmussen J. Langerman H. (2019). Alzheimer's disease - why we need early diagnosis. Degener Neurol Neuromuscul Dis 9, 123–130. doi: 10.2147/DNND.S228939, PMID: 31920420 PMC6935598

[ref78] Reppert S. M. Weaver D. R. (2002). Coordination of circadian timing in mammals. Nature 418, 935–941. doi: 10.1038/nature00965 12198538

[ref79] Roh J. H. Jiang H. Finn M. B. Stewart F. R. Mahan T. E. Cirrito J. R.. (2014). Potential role of orexin and sleep modulation in the pathogenesis of Alzheimer's disease. J. Exp. Med. 211, 2487–2496. doi: 10.1084/jem.20141788, PMID: 25422493 PMC4267230

[ref80] Rub U. Del Tredici K. Schultz C. Thal D. R. Braak E. Braak H. (2001). The autonomic higher order processing nuclei of the lower brain stem are among the early targets of the Alzheimer's disease-related cytoskeletal pathology. Acta Neuropathol. 101, 555–564. doi: 10.1007/s004010000320, PMID: 11515783

[ref81] Saper C. B. (2013). The central circadian timing system. Curr. Opin. Neurobiol. 23, 747–751. doi: 10.1016/j.conb.2013.04.004, PMID: 23706187 PMC3758384

[ref82] Saulea G. Hriscu M. Vidrascu N. Baciu I. (1998). Influence of bilateral olfactory bulbectomy on the circadian rhythm of phagocytic activity and phagocytic response in mice. Rom. J. Physiol. 35, 313–318. PMID: 11061330

[ref83] Scheyer O. Rahman A. Hristov H. Berkowitz C. Isaacson R. S. Diaz Brinton R.. (2018). Female sex and Alzheimer's risk: the menopause connection. J. Prev Alzheimers Dis. 5, 225–230. doi: 10.14283/jpad.2018.34, PMID: 30298180 PMC6198681

[ref84] Schonheit B. Zarski R. Ohm T. G. (2004). Spatial and temporal relationships between plaques and tangles in Alzheimer-pathology. Neurobiol. Aging 25, 697–711. doi: 10.1016/j.neurobiolaging.2003.09.009, PMID: 15165691

[ref85] Sengoku R. (2020). Aging and Alzheimer's disease pathology. Neuropathology 40, 22–29. doi: 10.1111/neup.12626, PMID: 31863504

[ref86] Sheehan P. W. Musiek E. S. (2020). Evaluating circadian dysfunction in mouse models of Alzheimer's disease: where do we stand? Front. Neurosci. 14:703. doi: 10.3389/fnins.2020.00703, PMID: 32733196 PMC7358444

[ref87] Silva M. V. F. Loures C. M. G. Alves L. C. V. de Souza L. C. Borges K. B. G. Carvalho M. D. G. (2019). Alzheimer's disease: risk factors and potentially protective measures. J. Biomed. Sci. 26:33. doi: 10.1186/s12929-019-0524-y, PMID: 31072403 PMC6507104

[ref88] Small S. A. Duff K. (2008). Linking Abeta and tau in late-onset Alzheimer's disease: a dual pathway hypothesis. Neuron 60, 534–542. doi: 10.1016/j.neuron.2008.11.007, PMID: 19038212 PMC2692134

[ref89] Spilman P. Podlutskaya N. Hart M. J. Debnath J. Gorostiza O. Bredesen D.. (2010). Inhibition of mTOR by rapamycin abolishes cognitive deficits and reduces amyloid-beta levels in a mouse model of Alzheimer's disease. PLoS One 5:e9979. doi: 10.1371/journal.pone.0009979 20376313 PMC2848616

[ref90] Sterniczuk R. Dyck R. H. Laferla F. M. Antle M. C. (2010). Characterization of the 3xTg-AD mouse model of Alzheimer's disease: part 1. Circadian changes. Brain Res 1348, 139–148. doi: 10.1016/j.brainres.2010.05.013, PMID: 20471965

[ref91] Takeuchi S. Shimizu K. Fukada Y. Emoto K. (2023). The circadian clock in the piriform cortex intrinsically tunes daily changes of odor-evoked neural activity. Commun Biol 6:332. doi: 10.1038/s42003-023-04691-8, PMID: 36973364 PMC10043281

[ref92] Todd W. D. Venner A. Anaclet C. Broadhurst R. Y. De Luca R. Bandaru S. S.. (2020). Suprachiasmatic VIP neurons are required for normal circadian rhythmicity and comprised of molecularly distinct subpopulations. Nat. Commun. 11:4410. doi: 10.1038/s41467-020-17197-2, PMID: 32879310 PMC7468160

[ref93] Tranah G. J. Blackwell T. Stone K. L. Ancoli-Israel S. Paudel M. L. Ensrud K. E.. (2011). Circadian activity rhythms and risk of incident dementia and mild cognitive impairment in older women. Ann. Neurol. 70, 722–732. doi: 10.1002/ana.22468, PMID: 22162057 PMC3244839

[ref94] van der Flier W. M. Pijnenburg Y. A. Fox N. C. Scheltens P. (2011). Early-onset versus late-onset Alzheimer's disease: the case of the missing APOE varepsilon4 allele. Lancet Neurol. 10, 280–288. doi: 10.1016/S1474-4422(10)70306-9, PMID: 21185234

[ref95] Vilensky J. A. (2014). The neglected cranial nerve: nervus terminalis (cranial nerve N). Clin. Anat. 27, 46–53. doi: 10.1002/ca.22130 22836597

[ref96] Vinkers C. H. Breuer M. E. Westphal K. G. Korte S. M. Oosting R. S. Olivier B.. (2009). Olfactory bulbectomy induces rapid and stable changes in basal and stress-induced locomotor activity, heart rate and body temperature responses in the home cage. Neuroscience 159, 39–46. doi: 10.1016/j.neuroscience.2008.12.009, PMID: 19136045

[ref97] Wang J. L. Lim A. S. Chiang W. Y. Hsieh W. H. Lo M. T. Schneider J. A.. (2015). Suprachiasmatic neuron numbers and rest-activity circadian rhythms in older humans. Ann. Neurol. 78, 317–322. doi: 10.1002/ana.24432, PMID: 25921596 PMC4515161

[ref98] Wang D. Wu J. Liu P. Li X. Li J. He M.. (2022). VIP interneurons regulate olfactory bulb output and contribute to odor detection and discrimination. Cell Rep. 38:110383. doi: 10.1016/j.celrep.2022.110383, PMID: 35172159

[ref99] Warfield A. E. Gupta P. Ruhmann M. M. Jeffs Q. L. Guidone G. C. Rhymes H. W.. (2023). A brainstem to circadian system circuit links tau pathology to sundowning-related disturbances in an Alzheimer's disease mouse model. Nat. Commun. 14:5027. doi: 10.1038/s41467-023-40546-w, PMID: 37596279 PMC10439113

[ref100] Wilson R. S. Arnold S. E. Schneider J. A. Boyle P. A. Buchman A. S. Bennett D. A. (2009). Olfactory impairment in presymptomatic Alzheimer's disease. Ann. N. Y. Acad. Sci. 1170, 730–735. doi: 10.1111/j.1749-6632.2009.04013.x, PMID: 19686220 PMC2857767

[ref101] Xie L. Kang H. Xu Q. Chen M. J. Liao Y. Thiyagarajan M.. (2013). Sleep drives metabolite clearance from the adult brain. Science 342, 373–377. doi: 10.1126/science.1241224, PMID: 24136970 PMC3880190

[ref102] Yokoyama M. Kobayashi H. Tatsumi L. Tomita T. (2022). Mouse models of Alzheimer's disease. Front. Mol. Neurosci. 15:912995. doi: 10.3389/fnmol.2022.912995 35799899 PMC9254908

[ref103] Yuan M. Y. Chen Z. K. Ni J. Wang T. X. Jiang S. Y. Dong H.. (2020). Ablation of olfactory bulb glutamatergic neurons induces depressive-like behaviors and sleep disturbances in mice. Psychopharmacology 237, 2517–2530. doi: 10.1007/s00213-020-05552-6, PMID: 32445053

[ref104] Zhang J. Zhao Z. Sun S. Li J. Wang Y. Dong J.. (2022). Olfactory evaluation in Alzheimer's disease model mice. Brain Sci. 12:607. doi: 10.3390/brainsci12050607, PMID: 35624994 PMC9139301

